# Second-generation ethanol ( GII ). The contribution of CTBE

**DOI:** 10.1186/1753-6561-8-S4-O39

**Published:** 2014-10-01

**Authors:** Carlos EV Rossell, Jose GC Pradella, Sindelia F Azzoni, Sarita C Rabelo, Jaciane L Ienczak, Chanel M carli

**Affiliations:** 1 Laboratório Nacional de Ciência e Tecnologia do Bioetanol-CTBE, Campinas, SP, Brazil

## 

The **Brazilian Bioethanol Science and Technology Laboratory (CTBE) **one of the laboratories of **the Brazilian Center of Research in Energy and Materials (CNPEM) **has as one of its main goals contribute to the development of the process of obtaining second generation ethanol ( GII ) mainly from lignin cellulosic fractions of cane sugar.

To achieve this goal leads its own line of research, develop partnerships with other public and private institutions or acting as national laboratory offers its facilities of laboratories and process development pilot plant to researchers involved in projects to produce ethanol, energy and chemicals from lignocelluloses materials.

The activities of CTBE are based on research and development line that integrates the current process of obtaining first-generation ethanol ( GI ) of the sugars extracted from cane sugar coupled with the second generation process of deconstruction of lignocelluloses fractions (bagasse and straw), enzymatic hydrolysis of cellulose and fermentation of pentose and hexoses.

The production of ethanol GII although technically demonstrated, at the present time not achieved results that enable production at competitive costs.

The critical barriers to overcome to achieve this goal are:

• Pretreatment: efficient fractionation of lignocelluloses material for better recovery and hydrolysis of pentose and cellulignin.

• Hydrolases: Obtaining a more efficient and productive hydrolases complex;

• Enzymatic hydrolysis: optimization of hydrolysis of cellulose;

• Ethanol from pentose: development of pentose fermentation to ethanol.

The negative impact of these barriers on the process of obtaining second-generation ethanol will be discussed. The research lines proposed for overcome it and improve the performance of this process integrated with first generation ethanol will be presented.

